# Climate Considerations in Long-Term Safety Assessments for Nuclear Waste Repositories

**DOI:** 10.1007/s13280-013-0406-6

**Published:** 2013-04-26

**Authors:** Jens-Ove Näslund, Jenny Brandefelt, Lillemor Claesson Liljedahl

**Affiliations:** Svensk Kärnbränslehantering AB, Box 250, 101 24 Stockholm, Sweden

**Keywords:** Climate scenario, Permafrost, Ice sheet, Global warming, Climate change

## Abstract

For a deep geological repository for spent nuclear fuel planned in Sweden, the safety assessment covers up to 1 million years. Climate scenarios range from high-end global warming for the coming 100 000 years, through deep permafrost, to large ice sheets during glacial conditions. In contrast, in an existing repository for short-lived waste the activity decays to low levels within a few tens of thousands of years. The shorter assessment period, 100 000 years, requires more focus on climate development over the coming tens of thousands of years, including the earliest possibility for permafrost growth and freezing of the engineered system. The handling of climate and climate change in safety assessments must be tailor-made for each repository concept and waste type. However, due to the uncertain future climate development on these vast time scales, all safety assessments for nuclear waste repositories require *a range* of possible climate scenarios.

**Electronic supplementary material:**

The online version of this article (doi:10.1007/s13280-013-0406-6) contains supplementary material, which is available to authorized users.

## Introduction

Changes in climate and climate-related processes need to be addressed in assessments of long-term safety of nuclear repositories. Since climate system evolution is not predictable on time scales up to 1 million years (1 Myr), a broad range of possible future climate scenarios is necessary for the analysis of nuclear waste repository safety (SKB 2010, 2011). The uncertainty in future climate system evolution is due to incomplete knowledge of past climate evolution and (coupled) processes of the climate system. Furthermore, modeling of future climate evolution is associated with uncertainty due to initial state and model formulation uncertainty (e.g., Stainforth et al. 2005; Crucifix and Rougier 2009). Input data and assumptions made in the safety assessment modeling work are selected so that the results are pessimistic in terms of analyses of safety of the nuclear waste repositories.

Geological archives show that Earth’s climate has evolved from warm (interglacial) to cold (glacial) periods, the latter characterized by ice sheet growth in high northern latitudes and permafrost conditions in ice-free high-latitude regions. For the past 2 Myr, the climate in Fennoscandia has been dominated by cold conditions with permafrost and at times extensive ice sheets. Based on this knowledge, periods of cold climate cannot be excluded in the next 100 000 years (100 kyr) to 1 Myr and thus future climate scenarios including permafrost growth and ice sheet formation are included in the range used for safety assessments for nuclear waste repositories.

In the last two centuries, atmospheric greenhouse gas concentration has increased and is expected to continue to increase due to human activities. The global annual average near-surface temperature increased by 0.76 °C from 1850–1899 to 2001–2005 (IPCC 2007). Following peak emissions, the atmospheric CO_2_ concentration, and the associated global average warming, is expected to decline slowly but still remain elevated for tens of thousands of years (NRC 2011). On a geological time scale, climate cycles are driven by changes in insolation (i.e., solar radiation received at the top of the atmosphere) as a result of variations in Earth’s orbit around the Sun (Berger and Loutre 2002). Over the next 100 kyr, the amplitude of insolation variations will be small, considerably smaller than during the last 200 kyr. The combined effect of high atmospheric greenhouse gas concentrations and low-amplitude variations in insolation on the future climate evolution has been investigated with simplified models of the climate system. These studies suggest that the initiation of the next Northern Hemisphere glaciation may occur around 50 kyr after present (AP) (Berger and Loutre 2002) or even several hundreds of kyr AP (Archer and Ganopolski 2005). Based on these indications, a long period of warm climate cannot be excluded in the next 100 kyr to 1 Myr. Thus, future climate scenarios including extended warm periods also need to be included in the range used for safety assessments for nuclear waste repositories.

In safety assessments, climate scenarios are used mainly for three purposes: (i) as basis for the description of the repository site development (for instance the landscape-, shoreline-, and lake development), (ii) in the analysis of the probability for a radionuclide release caused by variations in climate-related processes (for instance by high isostatic pressures from ice sheet load, or through freezing of repository barriers during permafrost periods), and (iii) in the analysis of the consequences of a radionuclide release, if the safety assessment shows that a release could occur.

The purpose of this article is to give an overview of how future climate evolution is handled in SKB safety assessments (Kautsky et al. 2013). The article describes how the climate scenarios were derived for safety assessment of (i) the planned long-term repository for spent nuclear fuel (KBS-3 type) and (ii) the extension of the existing repository for short-lived low- and intermediate-level waste (“SFR”). Both analyses were performed for Forsmark, south-central Sweden, where the existing and planned repositories are/will be located. Differences in the handling of future climate scenarios, due to differences in the repository concept and waste type, are discussed.

## Waste Type and Repository Concept Considerations

Radioactive waste is categorized based on initial radioactivity level (high-, intermediate-, and low-level) and radioactive decay rate (long- and short-lived). The combination of these characteristics determines the time frame for potential harm to humans and the environment, which motivates the time frame for the long-term safety requirements formulated by Swedish regulatory authorities. SKB handles three categories of waste: (i) high-level, long-lived waste (i.e., spent nuclear fuel), (ii) low- and intermediate-level short-lived operational waste (from nuclear and other installations), and (iii) low- and intermediate-level long-lived waste (from decommissioning of nuclear installations), planned to be placed in three separate repositories. The safety assessment for the planned repositories for long-lived waste (e.g., the spent nuclear fuel repository) therefore covers a longer period (1 Myr) than the safety assessment for the existing (and planned extension of the) repository for low- and intermediate-level waste SFR (100 kyr).

In the context of climate development, there is a large difference between analyzing a total time period of 1 Myr and one of 100 kyr, especially in the light of the ongoing global warming. In a 1-Myr time perspective, glacial conditions could be regarded as typical for sites located in terrain that previously has been glaciated by Late Pleistocene (800–10 000 years before present, BP) ice sheets (e.g., Porter 1989). This picture is only somewhat affected by an initial period of global warming, as the effects of anthropogenic greenhouse gas emissions would have tapered off well before 1 Myr AP. However, if the period to assess ends at ~100 kyr AP, the total time assessed in a scenario with strong global warming could be dominated by the global warming effect.

The questions that need to be answered in the assessments of long-term safety also differ among different types of repository concepts and the characteristics of their specific waste types. To exemplify this, one may consider the question of repository freezing, which, in some cases, potentially can result in safety barrier functions not being maintained. Analysis of the potential for repository freezing, including the consequences of freezing, should therefore be included in safety assessments for repository sites that have been subject to permafrost in the past and at which permafrost may be expected in the future. For one repository type the main question might be if the repository will freeze at repository depth or not, whereas the precise timing of such an event has a subordinate role due to a very slow rate of decay of key radionuclides. This is the case for KBS-3 repositories in Fennoscandia, with repository depths planned to be c. 400–700 m down in crystalline bedrock. Other types of repositories may contain radionuclide inventories with more short-lived isotopes and with the repository located at a significantly shallower depth. This may be exemplified by the existing shallow SFR repository at Forsmark. For SFR, the main concern is the timing of the first possible future freezing event, and one has to assume that the entire repository would freeze during severe cold climate scenarios.

The above example shows that the way of handling climate and climate-related issues may have to be different in different safety assessments. Given that the main question is different in safety assessments for different repository types, one has to adopt different approaches and methods in the treatment of climate. This, in turn, has an effect on other coupled parts of the safety assessment, such as the analysis of ground water flow and chemistry as well as the development of the biosphere (cf. Lindborg et al. 2013).

## An Approach to Handle Climate and Climate-Related Processes in Safety Assessments

In assessments of post-closure repository safety, the overall approach to handling the uncertainty in future climate development is to construct a range of climate scenarios aiming at covering this uncertainty. The range of scenarios typically consists of (i) *examples* of possible future climate evolution based, e.g., on repetitions of conditions reconstructed for the last glacial cycle or on estimates using current knowledge of human-induced warming and (ii) *bounding cases* that might have a larger impact on repository safety than the examples. The latter scenarios include, for example, the thickest expected ice sheets or deepest expected permafrost for the assessment period. To make the examples and bounding cases realistic, they are often constructed so as to include the full range of Quaternary natural climate variability based on palaeoclimate information.

Even if one cannot predict climate on the very long time scales analyzed in safety assessments, one can estimate the extremes within which the climate may vary with reasonable confidence. This can be done based on knowledge of palaeoclimate variations and on inferred future climate change. Within these limits, characteristic climate-related conditions of importance for repository safety can be identified and represented as climate-driven process domains (Boulton et al. 2001), where such a domain is defined as a climatically determined environment in which a set of characteristic processes of importance for repository safety appear. In the following, these climate-driven process domains are referred to as climate domains. The climate domains relevant for Northern Hemisphere high-latitude regions, including Sweden are: (i) the temperate climate domain, (ii) the periglacial climate domain, and (iii) the glacial climate domain.

In the two safety assessments discussed here, the temperate climate domain is defined as regions without permafrost or the presence of ice sheets. It is dominated by a temperate climate in a broad sense, see SKB (2010). The temperate climate domain has the warmest climate of the three domains. Within the temperate climate domain, a site may also at times be submerged by the sea. Climates dominated by anthropogenic global warming, characterized by higher temperatures and generally more precipitation than at present in Fennoscandia, are also included in the temperate climate domain.

The periglacial climate domain is defined strictly as regions with permafrost but without the presence of ice sheets. In this cold climate domain, permafrost may occur in sporadic (less than 50 % spatial coverage), discontinuous (between 50 and 90 % coverage), or continuous form (more than 90 % coverage). Although true for most of the time, regions belonging to the periglacial climate domain are not necessarily the same as regions with a climate that supports permafrost growth. For example, at the end of a period with periglacial climate domain the climate may be relatively warm, not building or even supporting the presence of permafrost. Instead, permafrost may be diminishing. The above definition of the periglacial climate domain, based on permafrost presence rather than temperature, is motivated by the importance of frozen ground for the safety function of nuclear repositories. In this climate domain, a site may at times be submerged by the sea. In general, the periglacial climate domain is colder than the temperate climate domain and warmer than the glacial climate domain.

The glacial climate domain is defined as regions that are covered by glaciers or ice sheets. Within the glacial climate domain, the ice sheet may in some cases be underlain by sub-glacial permafrost. Areas belonging to the glacial climate domain may not necessarily have a climate that supports the growth of ice sheets. However, the glacial climate domain is the coldest of the three climate domains.

It is likely that all three climate domains will appear repeatedly at Forsmark in the coming 1 Myr, whereas there is a possibility that one or two of the climate domains could dominate the coming 100 kyr.

### The Deep Repository for Spent Nuclear Fuel

**Fig. 1:**
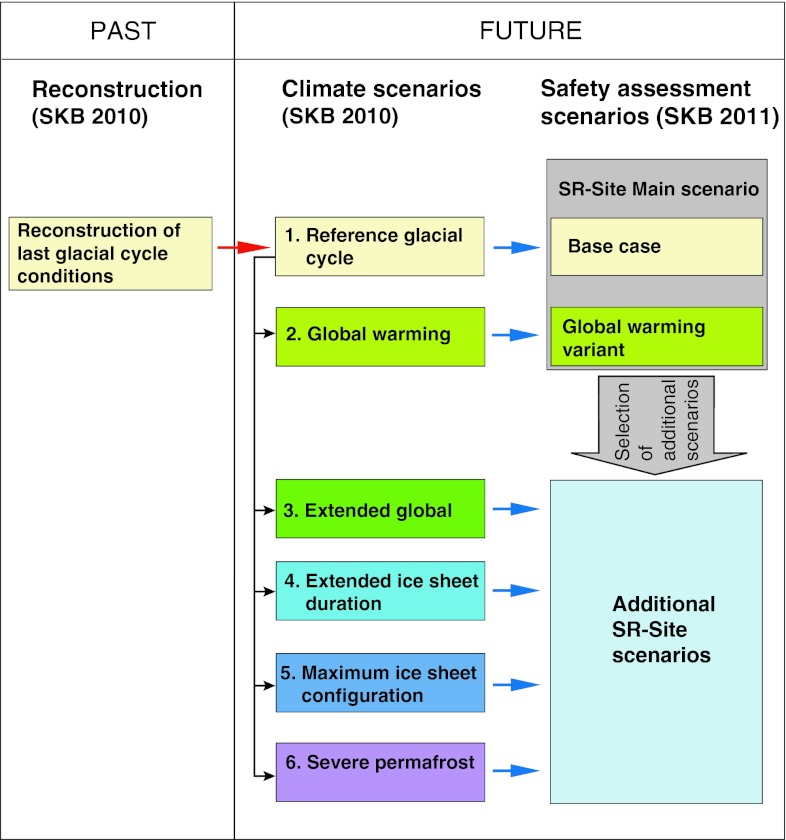
Relationship between the reconstruction of last glacial cycle conditions, the reference glacial cycle, the additional climate scenarios, and the corresponding safety assessment scenarios for the spent nuclear fuel repository. *Red arrow* indicates the choice of repeating the reconstructed last glacial cycle conditions for a future reference glacial cycle. *Black arrows* indicate modifications made to the reference glacial cycle to construct additional future climate scenarios to obtain a comprehensive coverage of possible climate developments of relevance for long-term safety. *Blue arrows* show which climate scenarios have been used to analyze which safety assessment scenario

Figure 1 shows an overview of the workflow for handling climate in the assessment for the spent nuclear fuel repository, starting with a reconstruction of last glacial cycle conditions, followed by the construction of future climate scenarios.

**Fig. 2:**
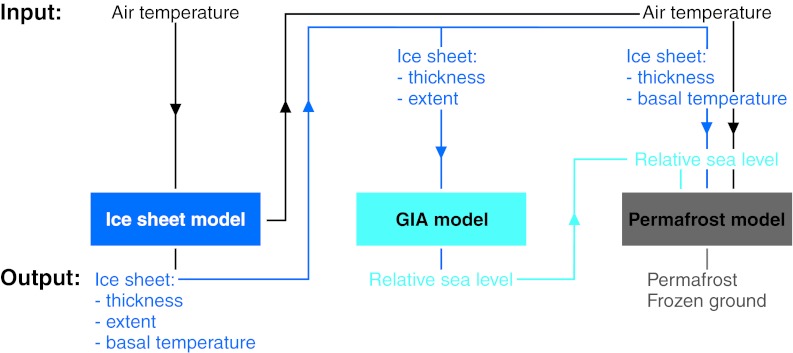
Example of coupled modeling performed for the safety assessment of the spent nuclear fuel repository. The model output was used to make a reconstruction of conditions for the last glacial cycle, in turn used for the construction of the reference glacial cycle. Only input and output data shared between the models used to generate the boundary conditions are shown

The climate domains are first used to describe a reference glacial cycle for the coming 120 kyr. The reference glacial cycle was constructed using a coupled modeling approach, in which data were shared between three models (Fig. 2). An ice sheet model was first used to simulate the growth and decay phases of the Weichselian ice sheet during the last glacial cycle. The ice-load history output was used as input to a global glacial isostatic adjustment (GIA) model simulating, for example, changes in shoreline elevation. Data for the Forsmark region were subsequently extracted from these two models and were used as input to site-specific simulations of permafrost development. In this way, a concordant reconstruction of last glacial cycle conditions for the Forsmark region was obtained. For a detailed description of the numerical modeling procedures, including details on models, input data, and assumptions, see SKB (2010). To handle uncertainties in input data in the various model simulations, pessimistic assumptions were typically made (SKB 2010). A systematic description of individual uncertainties or various combinations of uncertainties is often done; see for instance the sensitivity studies in the permafrost simulations in Hartikainen et al. (2010). Finally, the temporal reconstruction of ice sheet development, permafrost growth, and changes in shore line for the past 120 kyr were projected into the future to construct the reference glacial cycle.

The reference glacial cycle is not to be seen as a prediction of a future climate development at Forsmark, but as one example of an evolution of climate and climate-related processes fully dominated by natural climate variability in a 100 kyr and indeed 1-Myr time perspective.

Using a reference glacial cycle based on the last glacial cycle does not imply that glacial- and permafrost processes are regarded as more probable than processes related to warm climates for the next 100 kyr. To span the uncertainty range in future climate, five additional climate scenarios were defined based on: (i) knowledge of past changes in climate and environmental parameters, (ii) anticipated future climate change affected by anthropogenic action, and (iii) knowledge as to which processes are of importance for repository safety. In total, six scenarios of future climate development are described in the assessment for the spent nuclear fuel repository (Table 1).

**Table 1:** The six climate scenarios constructed and analyzed for the spent nuclear fuel repository, and their use in the safety assessment SR-Site

Climate scenario	Description	Basis on which the climate scenario was developed	Use in safety assessment
1. Reference glacial cycle	Repetition of conditions reconstructed for the last glacial cycle, including the Weichselian glaciation	Based on a reconstruction of ice sheet-, permafrost, and shore-line development for the last glacial cycle	Main safety assessment scenario—base case
2. Global warming	Moderate global warming. Longer period of initial temperate climate conditions than in 1	Based on a medium-level greenhouse gas emission scenario (IPCC emission scenario A1B). The maximum air temperature increase in the Forsmark region is 3.7 °C (uncertainty range 3.2–4.2 °C), occurring 2700 years AP. The air temperature returns to present conditions after c. 25 000 years	Main safety assessment scenario—variant
3. Extended global warming	Extensive and long-lasting global warming. Longer period of initial temperate climate conditions than in 1 and 2	Based on a high-level greenhouse gas emission scenario (IPCC emission scenario A2). The maximum air temperature increase in the Forsmark region is 6 °C (uncertainty range 3.9–6.5 °C), occurring 3000 years AP. The air temperature returns to present conditions after c. 50 000 years	Additional safety assessment scenario
4. Extended ice sheet duration	Longer period of glacial conditions that in 1	Based on an envisaged glacial cycle without ice-free conditions during a period corresponding to the interstadial reconstructed for the Middle Weichselian in the Reference glacial cycle	Additional safety assessment scenario
5. Maximum ice sheet configuration	Thicker ice sheet than in 1	Based on the largest ice sheet over Fennoscandia during the Quaternary period (past c. 2 Ma), i.e., the Saalian glaciations	Additional safety assessment scenario
6. Severe permafrost	Deeper permafrost than in 1	Based on the most pessimistic combination of assumptions for all parameters of importance to permafrost growth, including last glacial cycle air temperatures	Additional safety assessment scenario

The six climate scenarios were used as the basis for the construction of some of the safety assessment scenarios (Fig. 1). In the safety assessment, the reference glacial cycle is used to construct a main scenario, aiming at describing a reasonable evolution of the repository system and its environment. There are two variants of the main scenario: (i) a base case, comprising the reconstruction of climate and climate-related processes as described in the reference glacial cycle, i.e., the repetition of reconstructed last glacial cycle conditions and (ii) a global warming variant, defined from the global warming climate scenario.

To exemplify the use of the complementary climate scenarios, the maximum ice sheet configuration climate scenario is described here. The canisters containing the spent nuclear fuel must withstand the hydrostatic pressure induced by the overlying ice sheet thickness, together with the pressure from the overlying bedrock and the bentonite buffer swelling pressure. The effect of ice sheet thicknesses greater than those during the last glacial cycle is analyzed using information from the maximum ice sheet configuration scenario. This scenario is constructed by considering the largest and thickest ice sheet that occurred over Fennoscandia during the past 2 Myr, i.e., the Late Saalian ice sheet (c. 180–130 kyr BP) that existed toward the end of the penultimate glacial period. Also, the largest ice sheet thickness found on earth today, in East Antarctica, is discussed in the analysis. The maximum ice sheet configuration climate scenario comprises a bounding case in terms of maximum ice sheet thickness and hydrostatic pressure at repository depth.

**Fig. 3:**
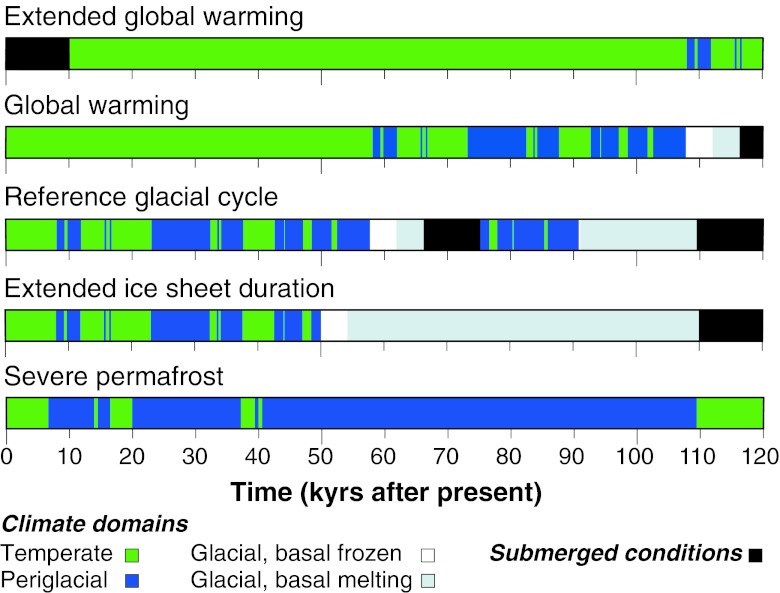
Future climate scenarios for the safety assessment of a high-level waste repository. The development of climate and climate-related processes (ice sheet growth, permafrost development, shore-line changes) are depicted by successions of climate domains (see text for explanation). The level of detail in the climate developments is obtained from the last glacial cycle conditions and reflects natural climate variability. The climate scenarios were used as basis for the analysis of long-term safety of the planned repository for spent nuclear fuel in Forsmark, Sweden (SKB 2010, 2011). Note that one of the additional climate scenarios, with the maximum ice sheet configuration and thickness, is not depicted in the figure but could be said to fit within the development shown in the *extended ice sheet duration* scenario

The climate scenarios constructed for the assessment for the spent nuclear fuel repository are displayed in Fig. 3. To cover the full 1-Myr time scale to be analyzed in this assessment, the reference glacial cycle was repeated seven additional times. The global warming scenario contributes with a variant of this development for the first 120 kyr. The longest period of temperate climate conditions for the coming 120 kyr, including an initial period with the warmest and wettest climate conditions, highest sea level, as well as the longest period of groundwater formation from precipitation, is found in the extended global warming scenario. The most extended period of periglacial climate conditions, including the deepest expected permafrost at Forsmark, is found in the severe permafrost scenario. The longest period of glacial conditions, and associated period of groundwater formation from glacial melt water, is found in the extended ice sheet duration scenario. The maximum future ice sheet thickness, resulting in the largest increase in hydrostatic pressure at repository depth, is found in the maximum ice sheet configuration scenario.

The six climate scenarios together cover the range within which climate and climate-related conditions of importance for long-term repository safety for a KBS-3 repository for spent nuclear fuel is expected to vary on a 100 kyr and 1-Myr time scale.

### The Shallow Repository for Short-Lived Low- and Intermediate-Level Waste

The ongoing safety assessment for the existing repository for short-lived low- and intermediate-level operational waste (“SFR”) in Forsmark, and the planned extension of this repository, cover 100 kyr. This repository differs from the planned spent nuclear fuel repository in several aspects. SFR is located at a shallow depth (c. 60 m) and the main part of the repository layout is today covered by the Baltic Sea. Furthermore, the radioactivity of the waste will decline significantly in the coming 10 kyr. The more detailed timing of climate events over the coming ten thousands of years is, therefore, of higher importance for the safety assessment, which was not the case for the deep geological repository for spent nuclear fuel. As freezing to SFR repository depth cannot be excluded in the next 100 kyr, and freezing may damage the SFR concrete barriers, an effort was made to construct plausible future climate scenarios with emphasis on the expected first occurrence of cold climate conditions.

In the climate work undertaken for SFR, all relevant climate scenarios from the assessment of the spent nuclear fuel repository were selected as a starting point: the global warming, extended global warming, and reference glacial cycle climate scenarios. Given the different character of the climate questions to be answered, the climate scenarios were reworked to better constrain the possible timing of the first period of periglacial conditions and permafrost in Forsmark. The specific studies performed to this end include: (i) the potential for permafrost in south-central Sweden in the next 60 kyr, (ii) ocean circulation response to increased atmospheric greenhouse gas concentration, (iii) future sea level change related to global warming, and (iv) palaeoclimate information on the initiation and characteristics of cold phases following the Eemian interglacial. The details of these studies are provided in the Appendix of the Electronic Supplementary Material.

## Discussion

Three climate scenarios that were included in the assessment of the spent nuclear fuel repository were not included in the assessment for the low- and intermediate-level repository: (i) the extended ice sheet duration scenario, (ii) the maximum ice sheet configuration scenario, and (iii) the severe permafrost scenario. These scenarios were all designed as bounding cases for parameters of importance specifically for high-level waste KBS-3 repository. The extended ice sheet duration scenario was used to analyze the effects a long periods of dilute ground water formation, which could affect, for example, bentonite buffer stability and groundwater oxygen content at repository depth. The maximum ice sheet configuration scenario was constructed to study the KBS-3 copper canister integrity under maximum hydrostatic pressures from large ice sheet loads, whereas the severe permafrost scenario was designed to test whether or not freezing could occur down to the repository depth of 450 m.

However, for the repository concept of SFR, with concrete barriers situated at shallow depth (60 m), the first process that could jeopardize repository safety is degradation of the concrete barrier by freezing. If freezing of the barriers was to occur within the coming few tens of thousands of years, the radionuclide inventory in SFR would still contain high levels of, e.g., ^14^C, which in case of a malfunctioning repository could significantly contribute to the risk calculated in the safety assessment. This risk would be considerably smaller if the barriers freeze later in the assessment period as the ^14^C radionuclide has a relatively short half-life (5.73 kyr). The first freezing of the SFR repository could occur during a future period of cold and dry climate conditions with permafrost developing at the Forsmark site, a situation expected to take place well before future ice sheet overriding of the site. To describe a climate scenario with an expected timing of first periglacial period, with respect to, e.g., future insolation variations and the present and possible future concentrations of atmospheric greenhouse gases, a new climate scenario called the early periglacial climate scenario was included in the assessment of the low- and intermediate-level waste repository (SKB 2013). This scenario differs from the severe permafrost scenario in the assessment for the spent nuclear fuel repository, as the latter was constructed to study maximum expected permafrost depths, rather than the timing of the first permafrost development at Forsmark.

**Fig. 4:**
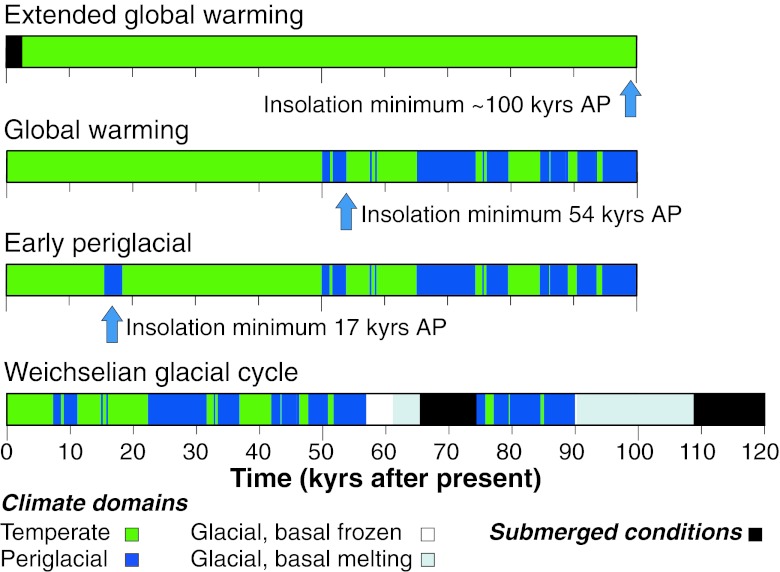
Climate scenarios for the assessment of long-term safety for a shallow repository for short-lived low- and intermediate-level waste (SKB 2013). The example is from the SKB safety assessment for the SFR repository located in Forsmark

There are large uncertainties in future climate development, uncertainties that, for the purpose of the assessment of SFR, are reasonably well covered by the global warming, extended global warming, and early periglacial climate scenarios (Fig. 4; SKB 2013). These scenarios all describe possible future developments that one could expect from the present scientific knowledge. Although not describing an expected future climate evolution, the Weichselian glacial cycle scenario is included to cover remaining uncertainties in understanding of the climate system.

None of the climate scenarios constructed for the safety assessments should be regarded as single stand-alone predictions of future climate development. Instead, the combination of all scenarios within each safety assessment covers the uncertainties in future development of climate and climate-related processes relevant for the specific repository type and waste. That is, each of these two sets of climate scenarios is based on the processes and questions that have been identified as important specifically for these two repository concepts. Other waste types with other repository concepts, such as the planned repository for long-lived low-level waste in Sweden, could require yet other variants of handling climate and climate-related processes.

## Conclusions

Two safety assessments performed for the Forsmark site in south-central Sweden are described in this article: one assessment for a deep geological repository for spent nuclear fuel, and one for a repository for short-lived low- and intermediate-level waste. Differences in the climate considerations in these assessments, depending on repository concept, activity and radioactive decay rate of the nuclear waste, and site-specific characteristics, are described. For each repository, a range of possible future climate scenarios is defined to span the uncertainty range for climate-related processes of importance for this specific repository.

For the planned deep geological repository for spent nuclear fuel in Forsmark, with the safety assessment covering a period of 1 Myr and with many radionuclides being very long-lived, the climate scenarios range from cases with high-end global warming for the coming 100 kyr, through cases with maximally deep permafrost, to cases with maximally large ice sheets during full glacial conditions. The latter scenarios are needed even if we are heading into a non-historical-analog situation with strong global warming, as the effects of global warming, regardless of its intensity, will have tapered off well before the end of the 1 Myr assessment period.

For the existing shallow SFR repository for short-lived low- and intermediate-level waste, with maximum potential radiological impacts from key radionuclides occurring in the coming few tens of thousands of years, and a total time to analyze restricted to c. 100 kyr, more focus needs to be put on the expected climate evolution over the coming tens of thousands of years. Here, the possibility of future periglacial conditions, and associated processes, such as permafrost growth and freezing of the shallow repository, are of prime importance compared to scenarios with subsequent ice sheet coverage.

To conclude, all safety assessments for repositories for nuclear waste, regardless of waste type and repository concept, require a range of possible future climate scenarios to cover the large uncertainty that exists in future climate development on the 100-kyr and 1-Myr time scales typically analyzed in such assessments.

## Electronic Supplementary Material

Below is the link to the electronic supplementary material.

Supplementary material 1 (PDF 251 kb)
