# Supplementary material for: Climate Considerations in Long-Term Safety Assessments for Nuclear Waste Repositories
Source: Ambio. 2013 Apr 26;42(4):393–401. doi: 10.1007/s13280-013-0406-6 (PMC3636373; doi:10.1007/s13280-013-0406-6)
Supplement: Supplementary file 1 — Supplementary material 1 (PDF 251 kb) [file 13280_2013_406_MOESM1_ESM.pdf]

Electronic Supplementary Material

Climate Considerations in Long-term Safety Assessments for Nuclear Waste Repositories

Jens-Ove Näslund, Jenny Brandefelt, Lillemor Claesson Liljedahl

APPENDIX

The potential for permafrost in south-central Sweden in the next 60 kyr

To investigate the potential for cold climate conditions without ice sheet growth in Forsmark over the coming 60 kyr, a combined climate- and permafrost modeling study was made. Minima in summer insolation at high northern latitudes will occur around 17, 54, and 100 kyr AP (e.g. Berger and Loutre 2002). The absolute minimum over the assessment period occurs 54 kyr AP, which is why this project is focused on the next 60 kyr rather than the complete 100 kyr assessment period.

To investigate the potential for cold climate conditions during periods of minimum northern high-latitude summer insolation, the Earth system model of intermediate complexity (EMIC) LOVECLIM (Driesschaert 2005; Driesschaert et al. 2007) and the state-of-the-art coupled atmosphere-ocean general circulation model (AOGCM) CCSM4 (Gent et al. 2011) were utilized. These models include atmospheric, oceanic, sea ice, land surface, and vegetation (LOVECLIM) of the global carbon–nitrogen cycle (CCSM4) components. Ice sheet dynamics however is not represented in any of the models.

In order to span the possible combinations of future orbital variations and atmospheric CO_2 concentration, a number of simulations were performed with LOVECLIM. All simulations were set up with constant-in-time atmospheric CO_2 concentration and with either a constant-in-time or time-varying latitudinal and seasonally varying insolation distribution. To estimate the uncertainty in

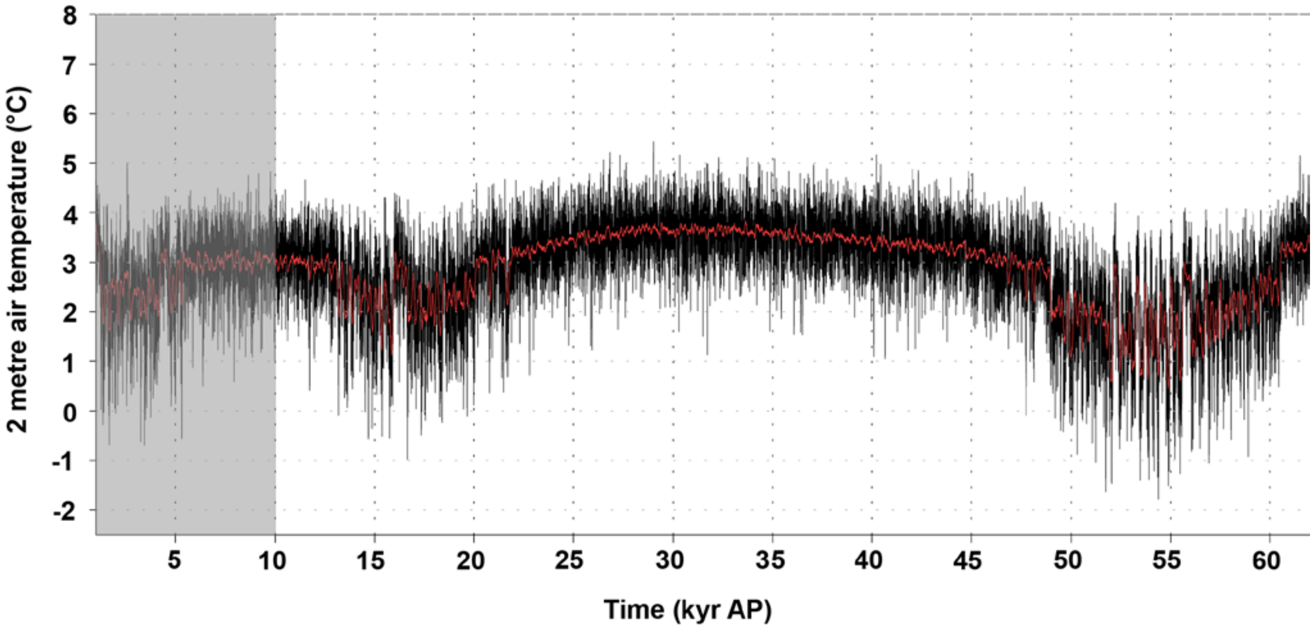

Fig. S1 Annual average air temperature for the Forsmark region for the coming 60 000 years simulated with the LOVECLIM climate model (black; from Brandefelt et al. 2013). The possibility of cold climate conditions in south-central Sweden in response to future variations in the latitudinal and seasonal distribution of insolation was studied under assumption of a very low constant atmospheric CO_2 concentration (200 ppmv). 100-year running-mean values are also displayed (red). The results clearly show colder climate conditions in the periods of low summer insolation at high northern latitudes during 17 and 54 kyr AP, and also that climate varies considerably during these periods. Note that the atmospheric CO_2 concentration is expected to exceed 280 ppmv for the coming 10 kyr, cf. Archer et al. (2009), because of the present high CO_2 level (392 ppm in 2011 AD) and the very slow loss of carbon from the atmosphere to terrestrial and marine 'sinks'. For the initial period, the expected future CO_2 concentration is thus in contrast to the very low constant CO_2 concentration assumed in this simulation. Therefore, the simulated cold temperatures for the initial period (for the coming c. 10 kyr) are, as expected, too cold and not realistic (grey area in the figure). For details on the climate model simulation, see Brandefelt et al. (2013). In order to investigate if the air temperatures during the cold periods at 17 and 54 kyr AP are low enough for permafrost development and freezing of SFR repository structures, air temperature data from the climate model were subsequently used as input to site-specific 2D permafrost simulations. In this analysis, several other pessimistic assumptions, besides from a low CO_2 level, and thorough considerations of uncertainties were also required

the LOVECLIM results, two simulations were performed with CCSM4.

As an example, Figure S1 shows the simulated annual average near-surface air temperature in the Forsmark region over the coming 60 kyr assuming a low atmospheric CO_2 concentration of 200 ppmv.

The influence of the variation in solar insolation is clearly seen in the figure with minima in air temperature coinciding with the occurrence of insolation minima around 17 and 54 kyr AP. Since the atmospheric CO_2 concentration is expected to remain above the pre-industrial 280 ppmv for the next 10 kyr or longer (e.g., Archer et al. 2009), the resulting air temperatures for the initial c. 10 kyr are lower than expected.

To investigate the potential for frozen ground and freezing of repository concrete barriers during the cold climate periods around 17 and 54 kyr AP, the climate model results were used as input to site-specific 2D permafrost model simulations. The permafrost model was similar to the one used for the permafrost simulations performed for the assessment of the spent nuclear fuel repository (Hartikainen et al. 2010), see Brandefelt et al. (2013).

Ocean circulation response to increased atmospheric greenhouse gas concentration

The oceanic meridional overturning circulation (MOC) is sensitive to changes in the density structure of the ocean. The density structure is expected to change in the future due to increasing atmospheric temperatures.

To assess the potential of cold climate in Fennoscandia due to regional cooling associated with changes in Atlantic oceanic northward heat transport, a literature review was performed (SKB 2013). IPCC (2007) concludes that it is likely that the MOC will decrease, but very unlikely that the MOC will undergo an abrupt transition during the course of the 21st century. They conclude that it is too early at this stage to assess the likelihood of an abrupt change of the MOC beyond the 21st century, but the possibility cannot be excluded. IPCC (2007) further concludes that if an abrupt reduction in the MOC was to occur, Europe would still experience warming as compared to the current climate since the radiative forcing caused by increasing greenhouse gases would overwhelm the cooling associated with the MOC reduction.

More recent studies indicate that the coupled models used in IPCC (2007) may be biased towards a more stable MOC than the observed and that a MOC collapse may occur within a few centuries (e.g., Drijfhout et al. 2011).

Stouffer et al. (2006) analyzed the MOC response to idealized freshwater perturbations among models ranging from EMICs to AOGCMs. A freshwater flux of 0.1 Sv (10^5 m^3 s^{-1}) and a flux ten times as large (1.0 Sv) are applied for 100 years, leading to an average MOC reduction of 30% in the 0.1 Sv experiments and a complete collapse in all the 1.0 Sv experiments.

The corresponding annual average near-surface temperature decrease in the Forsmark region varies in the range -7ºC to 0 ºC for the complete collapse of the MOC. In combination with the recent past annual average near-surface temperature of +5ºC and a warming due to increased atmospheric CO_2 concentration in the range of 2-5ºC, the minimum annual average near-surface temperature in the Forsmark region resulting from a complete collapse of the MOC is estimated to be 0ºC. This temperature is not sufficient to produce frozen ground in the Forsmark region (Hartikainen et al 2010).

Future sea level change related to global warming

In order to determine the maximum duration of submerged conditions at the Forsmark site for the Extended global warming climate scenario, sea-level changes that are in line with the air temperature development for this climate scenario were reviewed (SKB 2013). The information was used to define the initial period of submerged conditions for the Extended global warming climate scenario.

The combined effect of the different processes that could contribute to sea-level rise, and the isostatic rebound at the Forsmark site, suggests that the SFR repository could continue to be submerged by the Baltic Sea for another 2500 years at maximum (SKB 2013). Such submerged conditions have an effect on landscape development, groundwater flow pattern and groundwater chemistry, all of which are important parameters in the assessment of long-term repository safety.

Palaeoclimate information on the initiation and characteristics of cold phases following the Eemian interglacial

To enhance knowledge on the characteristics of a transition from a warm (interglacial) climate to a cold (glacial) climate, two additional literature reviews were performed (Wohlfarth 2013; Helmens 2013). The alteration and climatological characteristics of cold and warm climate events during the transition from the penultimate interglacial (the Eemian) into the last glacial period (the Weichselian), which occurred c. 115-80 kyr BP, were analysed on the basis of the literature and combined with data from more recent studies of geological archives (Wohlfarth 2013; Helmens 2013).

Results from the above studies, together with information from many other scientific publications were used to construct four climate scenarios for the safety assessment of the SFR repository (Table S1). The four climate scenarios are also displayed in Figure 5 of the main text.

The Global warming and Extended global warming climate scenarios were taken from the safety assessment for the repository for spent nuclear fuel and updated with the latest knowledge on, e.g., future sea level change. Based on the literature and modeling studies performed, a complementary climate scenario, the Early periglacial climate scenario was included. This scenario represents the uncertainty in the lifetime of the human-induced global warming in combination with minimum summer high northern latitude insolation around 17 kyr AP.

The Weichselian glacial cycle climate scenario was adopted unchanged from the Reference glacial cycle climate scenario in the safety assessment for the repository for spent nuclear fuel. This scenario is included to show the effect of natural climate

Table S1 The four climate scenarios constructed and analyzed for the low- and intermediate level waste repository, and their use in the safety assessment

Climate scenario	Description	Basis on which the climate scenario was developed	Use in safety assessment
**1. Global warming**	Moderate global warming.	Based on a medium-level greenhouse gas emission scenario (IPCC emission scenario A1B). The maximum air temperature increase in the Forsmark region is 3.7 °C (uncertainty range 3.2-4.2 °C), occurring 2700 years after present. The air temperature returns to present conditions after c. 25 000 years.	Safety assessment scenario
**2. Extended global warming**	Extensive and long-lasting global warming. Longer period of initial temperate climate conditions than in 1.	Based on a high-level greenhouse gas emission scenario (IPCC emission scenario A2). The maximum air temperature increase in the Forsmark region is 6 °C (uncertainty range 3.9-6.5 °C), occurring 3000 years after present. The air temperature returns to present conditions after c. 50 000 years.	Safety assessment scenario or Less probable scenario
**3a. Early periglacial without freezing of repository at 17 kyr AP**	Occurrence of first periglacial period around 17 kyr AP, without freezing of SFR repository barriers	Timing based on first potential period with colder climate in south-central Sweden when taking into account known future variations in insolation due to Earth's orbial parameters. The scenario assumes no freezing of concrete barriers.	Safety assessment scenario
**3b. Early periglacial with freezing of repository at 17 kyr AP**	Occurrence of first periglacial period around 17 kyr AP, with freezing of SFR repository barriers	Timing based on first potential period with colder climate in south-central Sweden when taking into account known future variations in insolation due to Earth's orbial parameters. The scenario assumes freezing of concrete barriers.	What-if case or Less probable scenario
**4. Weichselian glacial cycle**	Repetition of reconstructed last glacial cycle conditions. What-if case.	Based on a reconstruction of ice sheet-, permafrost and shore-line development for the last glacial cycle. The scenario is included in order to cover for remaining uncertainties in the understanding of the climate system, and to illustrate the effect of an ice sheet covering the repository.	What-if case

variability, as well as ice sheet coverage over the Forsmark site. Since permafrost growth in south-central Sweden is not likely to occur in the coming 10 kyr, the timing of, e.g., the first period of permafrost in Forsmark starting at c. 7 kyr AP in the Weichselian glacial cycle scenario is not considered realistic in the safety assessment for the low- and intermediate-level waste repository.

REFERENCES

Archer, D., and A. Ganopolski. 2005. A movable trigger: Fossil fuel CO_2 and the onset of the next glaciation. *Geochemistry Geophysics Geosystems* 6: 1-7. doi: 10.1029/2004GC000891

Berger, A., and M. F. Loutre. 2002. An exceptionally long interglacial ahead? *Science* 297: 1287-1288.

Brandefelt, J., Q. Zhang, J. O. Näslund, and J. Hartikainen. 2013. The potential for permafrost in south-central Sweden in the next 60,000 years. Svensk Kärnbränslehantering AB, SKB TR-13-XX (in press), Stockholm, Sweden, Report, xx pp.

Driesschaert, E. 2005. Climate change over the next millennia using LOVECLIM , a new Earth system model including the polar ice sheets. PhD thesis. Louvain-la-Neuve, Belgium: Université Catholique de Louvain.

Driesschaert, E., T. Fichefet, H. Goosse, P. Huybrechts, I. Janssens, A. Mouchet, G. Munhoven, V. Brovkin, et al. 2007. Modeling the influence of Greenland ice sheet melting on the Atlantic meridional overturning circulation during the next millennia. *Geophysical Research Letters* 34: 1–5. doi: 10.1029/2007GL029516.

Gent, P. R., G. Danabasoglu, L. J. Donner, M. M. Holland, E. C. Hunke, S. R. Jayne, D. M. Lawrence, R. B. Neale, et al. 2011. The Community Climate System Model Version 4. *Journal of Climate* 24: 4973–4991. doi: 10.1175/2011JCLI4083.1.

Hartikainen, J., R. Kouhia, and T. Wallroth. 2010. Permafrost simulations at Forsmark using a numerical 2D thermo-hydro-chemical model. Svensk Kärnbränslehantering AB, SKB TR-09-17, Stockholm, Sweden, Report, 142 pp.

Helmens, K. 2013. The last interglacial-glacial cycle (MIS 5-2) re-examined based on long proxy records from central and northern Europe. Svensk Kärnbränslehantering AB, SKB TR-13-xx (in press), Stockholm, Sweden, Report.

IPCC. 2007. Climate change 2007: the physical science basis. Contribution of Working Group I to the fourth assessment report of the Intergovernmental Panel on Climate Change. Cambridge, UK: Cambridge University Press. Retrieved January 6, 2013, from http://www.ipcc.ch/pdf/assessment-report/ar4/syr/ar4_syr.pdf

SKB. 2013. Climate and climate-related issues for the safety assessment SR-PSU. Svensk Kärnbränslehantering AB, SKB TR-13-XX (in press), Stockholm, Sweden, Report.

Stouffer, R. J., J. Yin, J. M. Gregory, K. W. Dixon, M. J. Spelman , W. Hurlin, A. J. Weaver, M. Eby, et al. 2006. Investigating the causes of the response of the thermohaline circulation to past and future climate changes. *Journal of Climate* 19: 1365-1387.

Wohlfarth, B. 2013. A review of Early Weichselian climate (MIS 5d-a) in Europe. Svensk Kärnbränslehantering AB, SKB TR-13-XX (in press), Stockholm, Sweden.
